# Shrinking the Metabolic Solution Space Using Experimental Datasets

**DOI:** 10.1371/journal.pcbi.1002662

**Published:** 2012-08-30

**Authors:** Jennifer L. Reed

**Affiliations:** Department of Chemical and Biological Engineering, University of Wisconsin–Madison, Madison, Wisconsin, United States of America; University of Virginia, United States of America

## Abstract

Constraint-based models of metabolism have been used in a variety of studies on drug discovery, metabolic engineering, evolution, and multi-species interactions. These genome-scale models can be generated for any sequenced organism since their main parameters (i.e., reaction stoichiometry) are highly conserved. Their relatively low parameter requirement makes these models easy to develop; however, these models often result in a solution space with multiple possible flux distributions, making it difficult to determine the precise flux state in the cell. Recent research efforts in this modeling field have investigated how additional experimental data, including gene expression, protein expression, metabolite concentrations, and kinetic parameters, can be used to reduce the solution space. This mini-review provides a summary of the data-driven computational approaches that are available for reducing the solution space and thereby improve predictions of intracellular fluxes by constraint-based models.

This is an “Editors' Outlook” article for *PLOS Computational Biology*.

## Introduction

Genome-scale constraint-based metabolic models can be used to predict or describe cellular behaviors, such as growth rates, uptake/secretion rates, and intracellular fluxes. These models have been used for a variety of applications, involving studies on drug discovery [1], metabolic engineering [2], evolution [3], genome annotation [4]–[6], and multi-species interactions [7]–[10]. Constraint-based metabolic models are developed by integrating genomic, biochemical, and physiological information for an organism, in a process that has been recently reviewed [11]. Computational and database efforts facilitate the construction of such models by automating some of the steps in the development process; for example, mapping genes to biochemical reactions or adding/removing reactions based on physiological data [4], [5], [12]–[14].

The variables used in constraint-based models include the fluxes through transport and metabolic reactions, and model parameters include reaction stoichiometry, biomass composition, ATP requirements, and the upper and lower bounds for individual fluxes. A common misconception is that these metabolic models rely on detailed kinetic parameters; however, such kinetic parameters are not required and are generally absent from most constraint-based models. Because there are often more variables (i.e., fluxes) than equations, no unique solution exists. The large number of solutions that satisfy the model's constraints define the model's solution space, which can be queried using a number of approaches [15]. Most of these constraints-based approaches utilize optimization to identify a subset of solutions of interest from within the solution space that are predicted to be physiologically relevant. For example, flux balance analysis (FBA) is often used to identify flux distributions that maximize biomass yields [16].

**Figure 1:**
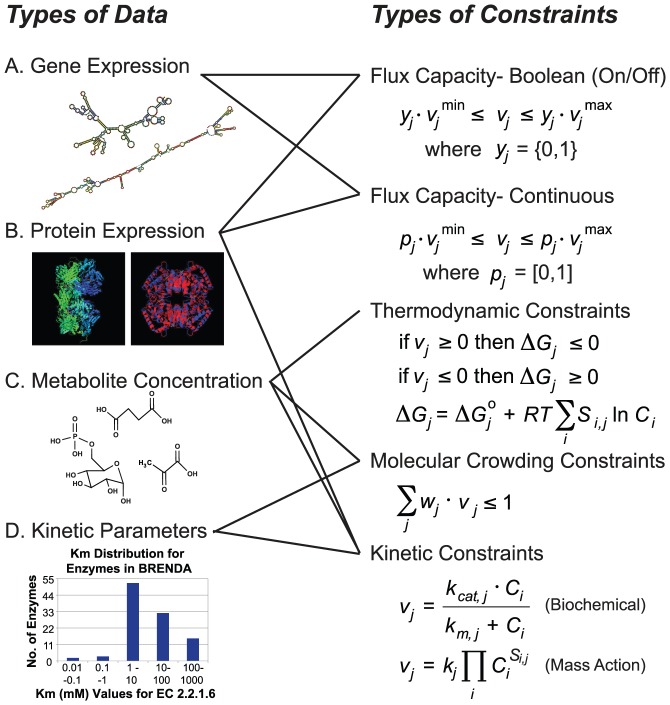
Experimental data and numerical constraints. Shown on the left are the different types of experimental data that can be accounted for in the models using the different types of metabolic flux constraints, shown on the right. Here, v denotes the metabolic fluxes, C denotes the metabolite concentrations, and k represents different kinetic parameters.

Given the non-uniqueness of constraint-based model solutions, a growing number of methods have focused recently on incorporating additional constraints to reduce the solution space and thereby improve the precision and accuracy of model predictions. This editorial reviews recent methods that utilize additional biological information (e.g., gene or protein expression, metabolite concentrations, and kinetic parameters) to further restrict metabolic fluxes, many of which are available in a variety of software packages [17]–[20]. A brief description of the standard constraints used in all constraint-based models is first presented, followed by a survey of how additional constraints have been included into models that often make use of additional types of experimental data (Figure 1).

## Standard Constraints

All constraint-based models use two types of fundamental constraints. Steady-state mass-balance constraints ensure that for each metabolite in the network the net production rate equals the net consumption rate. Additional inequality constraints are used to place restrictions or bounds on the values of individual fluxes based on measured rates (e.g., metabolite uptake/secretion rates) or reaction reversibility, where irreversible fluxes have a zero lower bound. Most models to date base reversibility on biochemical characterization of enzymes or consideration of network properties (e.g., no free ATP production). In standard models, none of these constraints limit metabolic fluxes based on metabolite, mRNA, or protein concentrations; however, a variety of additional constraints can be included based on thermodynamic, molecular crowding, gene expression, and regulatory and kinetic considerations.

## Thermodynamic Constraints

Thermodynamic constraints are used to place restrictions on the directionality of reactions by considering metabolite concentrations and Gibbs energies of formation. From thermodynamics, the change in Gibbs free energy for a reaction (ΔG) depends on the temperature, concentrations of substrates and products, and change in Gibbs free energies in a reference state (ΔG^O^). If a reaction is to proceed, the change in Gibbs free energy for a reaction must be negative. A few different approaches for incorporating these types of thermodynamic-based directionality constraints have been proposed. One of the first methods, network-embedded thermodynamic (NET) analysis, uses the directionality of reactions (based on pre-existing knowledge, experimental flux measurements, or constraint-based model results) to calculate ΔG or metabolite concentration ranges [21]. In NET analysis the reaction directions are determined a priori and a set of concentrations are found that are consistent with the thermodynamic constraints. However, analysis of thermodynamic constraints can also be done to identify reaction directionalities using specified metabolite concentrations (or concentration ranges). The results can then be used to limit the directionality of reactions in constraint-based models. Given the uncertainty in the Gibbs free energies of formation and metabolite concentrations, many reactions can operate in either direction and so probabilities can be used to assign uni-directional reactions [22]. Another approach, thermodynamic metabolic flux analysis (TMFA), directly imbeds the thermodynamic constraints into the models. TMFA uses integer variables to identify flux distributions that are consistent with thermodynamic constraints. In TMFA, fluxes and metabolite concentrations are variables in the models and constraints ensure that non-zero fluxes and ΔG values have opposite signs [23].

## Molecular Crowding Constraints

Recent efforts have used spatial constraints to place upper limits on a sum of fluxes, rather than individual fluxes. Molecular crowding constraints were first proposed by Beg et al. to restrict the total amount of enzyme that could be packed into a cell [24]. An upper limit on total enzyme volume was used and the volume of enzyme needed to sustain a given flux value was based on each enzyme's properties (e.g., kinetics and size). This molecular crowding constraint results in a restriction on the weighted sum of the fluxes, where the weights (w_j_) depend on an enzyme's volume and activity (less active, larger protein will have higher weights). Molecular crowding constraints have been used to predict cellular growth rates and acetate production in *Escherichia coli* [24], [25], to predict enzyme activities and metabolite concentrations in yeast [26], and to explain the Warburg effect of inefficient glucose catabolism in cancer cells [27]. Zhuang et al. recently extended this concept to impose limitations on the amount of enzymes that could reside in the cell membrane [28], thus placing restrictions on the weighted sum of fluxes through reactions that take place at the cell membrane. The authors investigated how this crowding constraint imposes a trade-off between glucose transport and respiratory pathways and showed that it was able to explain acetate production by *E. coli* under glucose aerobic conditions.

## Gene Expression Constraints

Gene expression is one of the most widely accessible measurements that can provide a global snapshot of a cell's metabolic state. A number of studies have compared constraint-based model flux predictions to expression data, to find consistencies and inconsistencies (e.g., [29]–[31]). For example, genes associated with reactions predicted to be essential for growth were found to have higher expression than those associated with reactions predicted to be inactive in *E. coli* [31]. On the other hand, fluxes predicted to be inactive in *Shewanella oneidensis* but whose genes were expressed identified pathways that were reducing biomass yields [29]. In these cases, the expression data are not used to help predict flux values, but instead are compared against flux predictions. As an alternative, a number of computational tools have been developed to integrate expression data into constraint-based models and restrict metabolic fluxes directly (Table 1).

**Table 1:** Comparison of methods for incorporating gene expression data.

Method	Thresholds	Description of Solutions
E-Flux	None	Finds solutions with fluxes whose upper limits are proportional to relative expression values
GIMME	One	Finds solutions with low flux through reactions associated with lowly expressed genes
Shlomi 2008	Two	Finds solutions with non-zero flux through reactions associated with highly expressed genes and zero flux through reactions associated with lowly expressed genes
MADE	None	Finds solutions whose gene's on/off states most closely match significant changes in gene expression across multiple conditions
Moxley 2009	None	Finds changes in flux values based on changes in gene expression values

Most current methods for incorporating gene expression data into the models compare gene expression levels in a single condition and disfavor fluxes through reactions that are associated with lowly expressed genes. The E-flux method uses gene expression values to set upper limits on metabolic fluxes, where reactions associated with more highly expressed genes will be allowed to take on higher flux values [32]. While E-flux places hard constraints on fluxes based on expression data, other methods instead use soft constraints that can be violated. GIMME tries to minimize the total inconsistency between fluxes and gene expression, where inconsistency depends on the flux value and the difference between a gene's expression value and a chosen threshold [33]. In this case, GIMME will try and reduce fluxes through reactions whose associated gene's expression falls below the threshold. Another method, developed by Shlomi et al. [34], tries to encourage flux through reactions whose associated genes are highly expressed and discourage flux through reactions whose associated genes are lowly expressed. With this method, high and low expression thresholds are chosen and used to assign reactions to high, low, or moderate groups. Using optimization, fluxes are then favored through reactions belonging in the high group and disfavored through reactions belonging to the low group.

All of these previous methods typically use expression data from a single condition to constrain fluxes. A more recent approach (MADE) uses expression data from multiple conditions (or a time-series) to identify patterns of increased/decreased expression based on significant changes in expression across conditions [18]. With MADE, the measured patterns of expression increases and decreases are used to find gene on/off patterns in the model across all conditions, where more significant expression changes are weighted more heavily. In another study, Moxley et al. used expression changes between two conditions to predict flux changes [35]. Using two global parameters they were able to accurately predict flux changes from gene expression changes using non-linear functions that account for metabolite-enzyme interaction densities.

## Transcriptional Regulatory Constraints

The methods described above for using gene expression-based constraints require expression data under the condition(s) of interest. In other words, to predict flux in a particular condition the methods would need gene expression data from that condition. Other methods can instead use models of transcriptional regulatory networks to predict the effects of transcriptional regulation on metabolic fluxes. In this case, integrated models of metabolism and regulation can predict metabolic fluxes under conditions (e.g., gene knockout mutants) for which gene expression data are not available. Transcriptional regulatory networks can be reconstructed from high-throughput data, such as gene expression, ChIP-chip, and genome sequencing datasets using a variety of approaches (reviewed in [36]–[38]). To date, two different types of approaches have been used to incorporate transcriptional regulatory constraints into genome-scale metabolic models. The first set of approaches used a Boolean (on/off) representation of transcriptional regulation, where Boolean rules are used to determine the state of transcription factors (active or inactive) and metabolic genes (expressed or not expressed). Based on the expression states of metabolic genes, the reactions in the metabolic network can (if necessary genes are expressed) or cannot (if necessary genes are not expressed) carry flux [39]. Analysis of these Boolean types of models can be done by solving the regulatory and metabolic models separately in an iterative fashion (rFBA) or simultaneously (SR-FBA) by introducing integer variables to represent the transcription factor/gene expression/reaction on/off states [40]–[42]. Not all regulation can be captured using a Boolean approach; for example, essential genes must always be on even though their expression may be regulated. To overcome this limitation, another type of approach has recently been used to formulate regulatory constraints based on a probabilistic regulatory model, where a continuous rather than a Boolean flux constraint is used. Here, the regulatory model predicts the probability that a given gene is expressed and this probability is used to weight the upper and lower limits that a metabolic flux can achieve [43]. The resulting model integrates both the metabolic and regulatory networks using a method called probabilistic regulation of metabolism (PROM).

## Kinetic Constraints

A variety of approaches have been developed to capture kinetic limitations in the models. These approaches involve constraining either the uptake/secretion rates using empirical rate laws that depend on extracellular concentrations or constraining intracellular fluxes using enzymatic rate laws that depend on intracellular and extracellular concentrations. Incorporating constraints on the uptake or secretion rates of metabolites often requires material balance equations for the bioreactor environment, in addition to the standard metabolic constraints for the cells. Empirical rate laws are found by fitting metabolite uptake/secretion rates to measured reactor concentrations. These rate laws are then used as additional constraints in the models. The resulting dynamic FBA (dFBA) models can then use bioreactor concentrations to constrain metabolic fluxes, which in turn affect the bioreactor concentrations. Feng et al. recently included rate laws for the uptake and secretion of organic acids into a genome-scale model for *S. oneidensis* to evaluate tradeoffs between maximizing growth and minimizing enzyme usage in batch culture [44]. Such empirical constraints have also been used to restrict uptake rates in co-culture models of environmental and industrial microbes [10], [45].

Traditional kinetic models already take into account the kinetic relationships between metabolic fluxes, metabolite, and protein concentrations. However, such detailed models are often available for only a few pathways in well-characterized organisms, such as *E. coli* and *Saccharomyces cerevisiae*, since the kinetic properties of their enzymes have been biochemically characterized. Databases, such as BRENDA [46] and SABIO-RK [47], contain an extensive collection of kinetic parameters assembled from the biochemical literature, and these in vitro estimates can be used to formulate kinetic constraints. While kinetic models exist for central metabolism and other isolated pathways, expanding these models to a genome scale is an active area of research [48]–[51]. Yizhak et al. recently developed an approach called IOMA [52], which uses kinetic expressions for a subset of enzymes to constrain metabolic fluxes. By incorporating multi-omics datasets using kinetic constraints for 11 reactions into an *E. coli* model, the authors were able to improve flux predictions in 23 gene deletion strains [52].

## Conclusions

As we continue to be able to measure intracellular levels of biological components with greater accuracy and precision, the need for computational approaches to integrate and analyze such large-scale datasets grows. As reviewed above, a variety of constraint-based approaches are available that use these types of datasets to reduce the solution space and improve model predictions of metabolic phenotypes. Over the coming years, more computational approaches for integrating individual and multiple types of experimental measurements will likely appear, as new biological measurement approaches are developed and more data becomes available. For example, we are likely to see integration of datasets into models of microbial communities, as multi-species models [8], [10], [53] and datasets become available. With recent advances in the ability to rapidly build genome-scale models [12], there will also be a need to design experiments whose results would best reduce the metabolic solution space. One of the future challenges is then to prioritize what types of data are important to measure and for which components. Other related questions need to be answered as well. How important is it to have absolute versus relative concentration measurements? What experimental precision is needed for different types of data? The answers to all of these questions will depend on both the biological hypotheses that are being investigated and the desired precision for predicted fluxes, which specifies how much the solution space needs to shrink.

**Author's Biography**

**Jennifer L. Reed** is an Assistant Professor in the Department of Chemical and Biological Engineering at the University of Wisconsin–Madison and a Project Lead at the Great Lakes Bioenergy Research Center. She is a recipient of an NSF CAREER award and a DOE Early Career award. She has been an Associate Editor for *PLOS Computational Biology* since 2010. She received her BS degree in Bioengineering: Biotechnology in 2000 and her PhD in Bioengineering in 2005 from the University of California, San Diego. She spent two years after her PhD as a Faculty Fellow at the University of California, San Diego, where she taught classes and conducted research in the Bioengineering Department. Her research interests are in the development and analysis of microbial metabolic and regulatory models for bioenergy, biotechnology, and health applications. Her research has included the development of genome-scale models for a variety of different microbes, including *Escherichia coli*, *Salmonella*, *Shewanella*, and Cyanobacteria species, as well as computational tools for metabolic engineering, model comparison, model refinement, and experimental design.
